# Sex difference in the incidence of stroke and its corresponding influence factors: results from a follow-up 8.4 years of rural China hypertensive prospective cohort study

**DOI:** 10.1186/s12944-019-1010-y

**Published:** 2019-03-25

**Authors:** Yali Wang, Yue Dai, Jia Zheng, Yanxia Xie, Rongrong Guo, Xiaofan Guo, Guozhe Sun, Zhaoqing Sun, Yingxian Sun, Liqiang Zheng

**Affiliations:** 1 Department of Clinical Epidemiology, Library, Department of Health Policy and Hospital Management, Shengjing Hospital of China Medical University, Shenyang, 110004 People’s Republic of China; 2 Department of Cardiology, the First Affiliated Hospital of China Medical University, Shenyang, 110001 People’s Republic of China; 3 Department of Cardiology, Shengjing Hospital of China Medical University, 36 Sanhao Street, Heping District, Shenyang, 110004 People’s Republic of China

**Keywords:** Stroke, Sex difference, Influence factors, Incidence

## Abstract

**Background:**

Few studies investigate sex difference in stroke incidence in rural China hypertensive population.

**Methods:**

A total of 5097 hypertensive patients aged ≥35 years (mean age, 56.3 ± 11.2 years; 43.8% men) were included in our analysis with a median follow-up 8.4 years in Fuxin county of Liaoning province in China. Cox proportional hazard models were used to analyze the association between the potential factors and incident stroke.

**Results:**

We observed 501 new strokes (310 ischemic, 186 hemorrhagic, and 5 unclassified stroke) during the follow-up. The overall incidence of stroke was 1235.21 per 100,000 person-years; for men, the rates were 1652.51 and 920.80 for women. This sex difference in all stroke can be explained by approximately 25% through age, systolic blood pressure, body mass index, low-density lipoprotein-cholesterol, current smoking, current drinking, antihypertensive drugs, education and physical activity. Subgroup analysis indicated that in hemorrhagic stroke this sex difference was more remarkable (63.89% can be explained).

**Conclusions:**

The incidence of stroke was higher in men than that in women and this difference was partly explained by several traditional cardiovascular risk factors.

## Background

Stroke is the second most common cause of death in the world [1], although some studies have found that the incidence and mortality of stroke has been decreased in some countries [2–5]. However, it has been the leading cause of death in China in recent years, particularly high in rural areas, and contribute to biggest social burden [6, 7]. A recent study which was conducted in 155 urban and rural centers in 31 provinces in China including 480,687 adults aged ≥20 years found that the age-standardized incidence of stroke was 246.8 per 100,000 person-years [6].

In the past 2 decades, China has experienced rapid health transitions and sociodemographic changes that have had an impact on the prevalence of common stroke risk factors. The prevalence of hypertension, smoking, diabetes and obesity has a great increase that might have affected the stroke incidence [6].

Sex differences in stroke incidence have been reported [7–13]. A 56-year follow-up prospective study from the Framingham Heart Study including 10,076 participants found that women had a higher stroke incidence above 85 years of age, lower at all other ages [12]. Another population-based study in Sweden found stroke incidence to be 60% lower for women than men at ages 55–64 years, but by the age of 75 years women had a 50% higher incidence than men [13]. However, most of these studies were conducted in Western countries and the data about the sex difference in stroke incidence in China was scarce. In addition, few studies have quantitatively investigated the potential factors that affect this disparity [7, 8]. And understanding this sex difference and its influence factors can help us prevention stroke better.

The stroke incidence was particularly higher in rural areas of China and hypertensive patients were at high risk of developing stroke. Therefore, we aimed to explore the sex difference in stroke incidence in rural areas of China among hypertensive patients.

## Methods

### Study population and study design

**Fig. 1:**
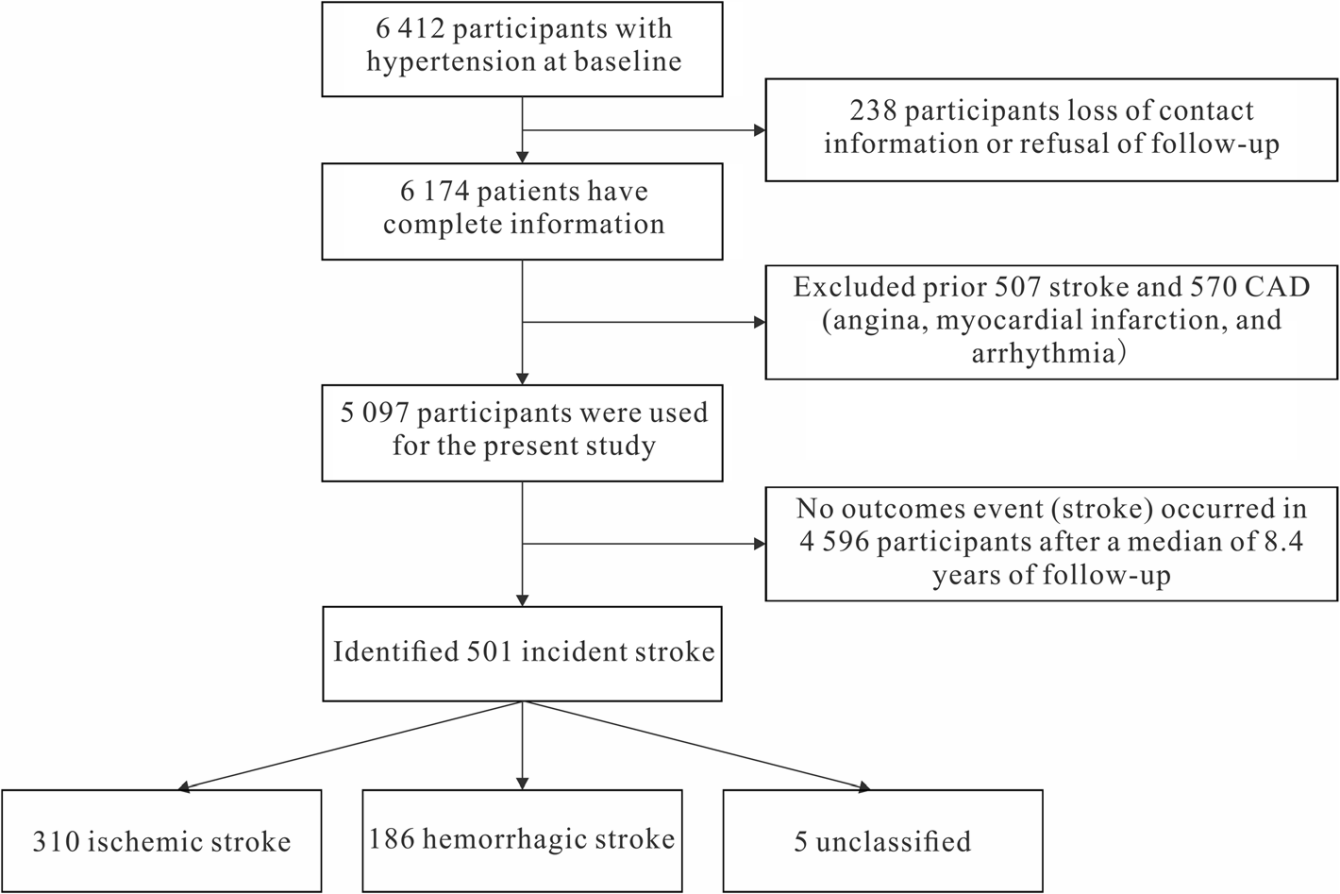
The study population inclusion and exclusion process. CAD: coronary artery disease

This is a large-scale epidemiological prospective study. From 2004 to 2006, a multistage, random cluster sampling process was performed to select a representative cohort of the rural population with hypertension aged ≥35 years from 50 rural villages in Liaoning Province. The detailed description is described elsewhere [14, 15]. All study participants were invited to return for follow-up: from January to June 2008; from July to October 2010; and from August to October 2014. Of the 6412 participants with hypertension at baseline, 238 lost follow-up due to loss of contact information or refusal of follow-up. 6174 patients completed at least one follow-up visit. We also excluded prior stroke (*n* = 507), CAD (angina, myocardial infarction, and arrhythmia) (*n* = 570), leaving 5097 hypertensive patients free from cardiovascular disease (CVD) for prospective analysis. The study population inclusion and exclusion process was illustrated in Fig. 1. The research protocol was approved by the China Medical University Research Ethics Committee, and written informed consent was formally obtained from all the participants or their guardians.

### Data collection and physical examinations at baseline

During the interview and examination, doctors performed a standard questionnaire to obtain lifestyle factors (current smoking, alcohol use). We converted the different varieties of wine already on the market into the corresponding grams of alcohol at different concentrations. Current drinking was defined as more than 1 drink/day for women and more than 2 drinks/day for men during the last year [16]. Current smoking was defined as smoking at least one cigarette per day and lasting for at least a year. Physical activity was classified three groups: low, moderate, and high. A detailed methodology was described elsewhere [17].

Body weight and height were measured with subjects wearing light clothing and without shoes. Body mass index (BMI) was calculated by the weight in kilograms divided by height in square meters. Blood pressure (BP) was measured with a checked electronic sphygmomanometer (Omron; Dalian, Liaoning, China), which had already been validated by the British Hypertension Society protocol [18]. BP was measured 3 times in the left arm after the subjects had been at rest in the sitting position for ≥5 min, and the mean value of the 3 measurements were used to determine the reported BP for that examination. Hypertension was defined as average SBP ≥140 mmHg or/and average DBP ≥90 mmHg or/and use of antihypertensive medications within the previous 2 weeks.

Participants were asked to fast for at least 12 h before blood collection. Blood chemical analyses were performed at a central, certified laboratory. Serum glucose, total cholesterol, low-density lipoprotein cholesterol (LDL-C), high-density lipoprotein cholesterol (HDL-C), and triglycerides were analyzed enzymatically on an Olympus AU640 autoanalyzer (Olympus, Kobe, Japan).

### Stroke assessment

Stroke events were defined the WHO Multinational Monitoring of Trends and Determinants in Cardiovascular Disease (MONICA) criteria [19, 20]: rapidly developing signs of focal (or global) disturbance of cerebral function lasting > 24 h (unless interrupted by surgery or death) with no apparent nonvascular cause. Hemorrhagic stroke was defined as stroke event with a diagnosis of subarachnoid hemorrhage or intracerebral hemorrhage, and ischemic stroke was defined as stroke event with a diagnosis of thrombosis or embolism. Transient ischemic attacks and silent brain infarctions (cases without clinical symptoms or signs) were not included, neither were events associated with trauma, hematologic disorders, or malignancy. The relevant information was obtained by direct reference to medical records by a single investigator. All materials were independently reviewed by the end-point assessment committee, whose members were all blinded to the study participants’ baseline risk factor information.

### Statistical analysis

Continuous variables are presented as the mean and standard deviation (SD) and are compared with Student’s t-test. Categorical variables are expressed as percentages and Pearson’s χ^2^ tests for independent proportions. The association between the influence factors and incident stroke was analyzed by the Cox proportional hazard models and calculated the hazard ratios (HR) and its corresponding 95% confidence interval (CI). We conducted a base model where only sex was included as the independent variable to simulate the difference in the incidence of stroke between men and women and obtained the HR of sex. Then we added each of those variables which showed a significant difference between the sex into the model and calculated the percentage of difference of HR for sex after adding the variable. The percentage of difference after adding a specified variable to the model was calculated using the following formula [21]:

$$ \left[\left({HR}_1-{HR}_2\right)/\left({HR}_1-1.0\right)\right]\times 100\% $$

Where HR_1_ represents HR of sex derived from the base model; and HR_2_ represents HR of sex after adding in the designated variable(s). All analyses were performed with IBM SPSS statistical software version 22.0. A *P* value less than 0.05 was accepted as statistically significant.

## Results

Table 1 shows the baseline characteristics of the study population. The mean age of the study population was 56.3 (SD 11.2) years and 2231 (43.8%) were men. Compared with men, women were more likely to have higher SBP levels and taking antihypertensive medications and they were less likely to smoking and drinking.

**Table 1:** The characteristics of the study population at baseline (*n* = 5097)

Characteristics ^a^	Men (*n* = 2231)	Women (*n* = 2866)	*P* Value ^b^
Age, (years)	57.21 ± 11.52	55.54 ± 10.97	<0.001
BMI,(kg/m^2^)	23.45 ± 3.01	24.19 ± 3.63	<0.001
SBP, (mmHg)	158.01 ± 20.41	161.06 ± 21.41	<0.001
DBP, (mmHg)	94.05 ± 11.97	93.91 ± 12.02	0.691
Blood glucose, (mmol/L)	5.70 ± 1.49	5.70 ± 1.86	0.896
Total cholesterol, (mmol/L)	5.17 ± 1.06	5.30 ± 1.04	<0.001
Triglyceride, (mmol/L)	1.65 ± 1.82	1.75 ± 1.30	0.030
Salt intake, (g/day)	16.7 ± 11.38	16.37 ± 12.49	0.341
LDL-C, (mmol/L)	2.72 ± 0.72	2.86 ± 0.73	<0.001
HDL-C, (mmol/L)	1.42 ± 0.33	1.44 ± 0.32	0.004
Current smoking, n (%)	1458 (65.4)	587 (20.5)	<0.001
Current drinking, n (%)	1290 (57.8)	213 (7.4)	<0.001
Antihypertensive drugs, n (%)	412 (18.5)	779 (27.2)	<0.001
Age, n (%)			
35–44 years	356 (16.0)	540 (18.8)	<0.001
45–54 years	595 (26.7)	849 (29.6)	
55–64 years	651 (29.2)	871 (30.4)	
≥ 65 years	629 (28.2)	606 (21.1)	
Education degree, n (%)			
Illiteracy and primary school	1006 (45.1)	1799 (62.8)	<0.001
Junior high school	1012 (45.4)	953 (33.3)	
High school and above	213 (9.5)	114 (4.0)	
BMI group, n (%)			
<24	1381 (61.9)	1460 (50.9)	<0.001
24–28	719 (32.2)	1052 (36.7)	
≥ 28	131 (5.9)	354 (12.4)	
Ethnicity, n (%)			
Han	1788 (80.1)	2289 (79.9)	0.955
Mongolian	411 (18.4)	537 (18.7)	
Other	32 (1.4)	40 (1.4)	
Physical activity, n (%)			
Low	671 (30.4)	1036 (36.5)	<0.001
Moderate	879 (39.8)	1077 (37.9)	
High	656 (29.7)	725 (25.5)	
History of family hypertension, n (%)			0.032
None of parents	1841 (82.5)	2297 (80.1)	
At least one parent	390 (17.5)	569 (19.9)	
History of diabetes, n (%)	13 (0.6)	27 (0.9)	0.149
History of hyperlipidemia, n (%)	128 (5.7)	169 (5.9)	0.810

LDL-C: low-density lipoprotein cholesterol. HDL-C:high-density lipoprotein cholesterol. BMI: Body mass index

SBP: systolic blood pressure. DBP: diastolic blood pressure

^a^:Population characteristics in the table are percentage or mean (standard deviation)

^b^:*P*-values were calculated by t test or chi-square test to compare differences within sex

**Fig. 2:**
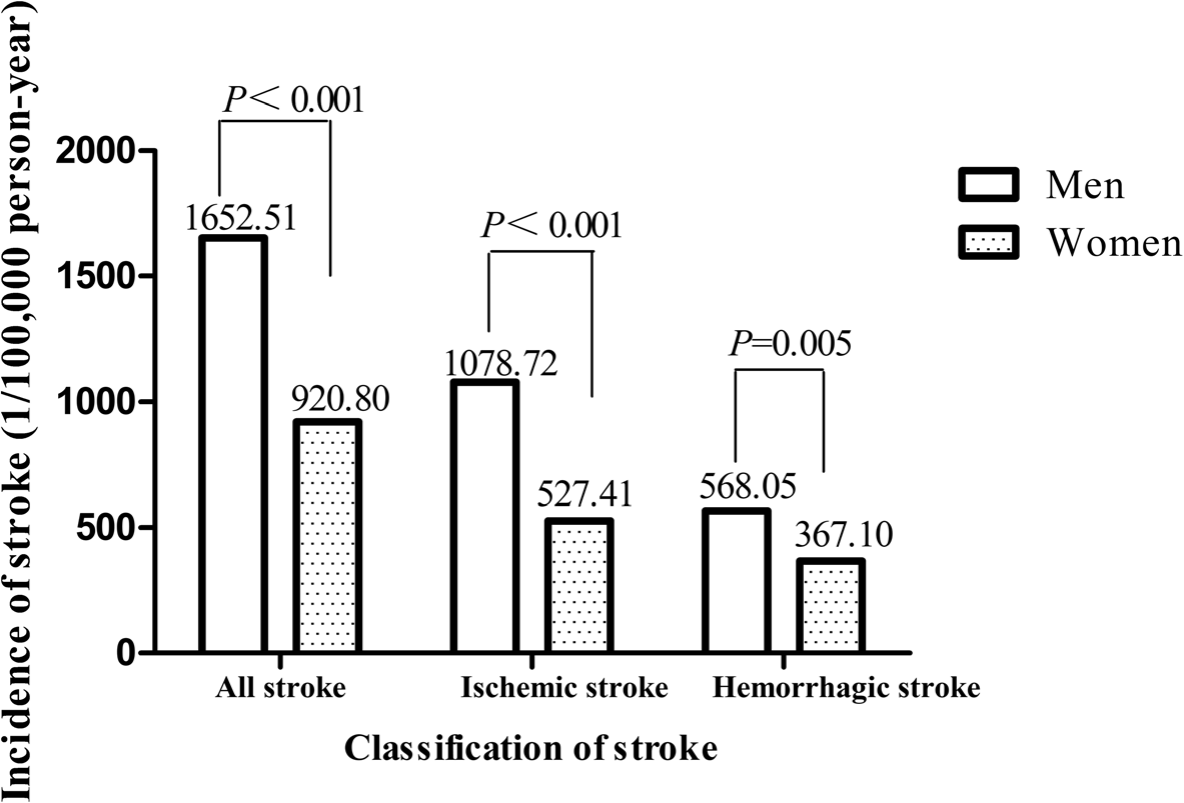
The incidence of stroke and its subtypes by sex

As illustrates in Table 2**,** during a median follow-up period of 8.4 years, we identified 501 incident stroke (310 ischemic, 186 hemorrhagic, and 5 unclassified). We followed 40,560 person-years in total and the overall incidence of stroke was 1235.21 per 100,000 person-years; for men, the rates were 1652.51 per 100,000 person-years and 920.80 per 100,000 person-years for women. Subgroup analysis shows that the incident rates of ischemic and hemorrhagic stroke were 1078.72 and 568.05 per 100,000 person-years in men and 527.41 and 367.10 per 100,000 person-years in women. The incidence of stroke and its subtypes were higher in men than that in women (Fig. 2).

**Table 2:** Incidence of stroke according to sex

Patients	Men	Women
Person-years of follow-up	17,428	23,132
All stroke		
Number	288	213
Incident rate per 100,000 person-years (95% CI)	1652.51 (1463.24–1841.80)	920.80 (797.71–1043.90)
Sex-adjusted HR (95% CI)	1.00(Referent)	0.55 (0.46–0.66)
Multivariable-adjusted ^a^ HR (95% CI)	1.00(Referent)	0.44 (0.35–0.55)
Ischemic stroke		
Number	188	122
Incident rate per 100,000 person-years (95% CI)	1078.72 (925.36–1232.10)	527.41 (434.07–620.70)
Sex-adjusted HR (95% CI)	1.00(Referent)	0.48 (0.38–0.61)
Multivariable-adjusted ^a^ HR (95% CI)	1.00(Referent)	0.43 (0.32–0.57)
Hemorrhagic stroke		
Number	99	87
Incident rate per 100,000 person-years (95% CI)	568.05 (984.10–1299.60)	367.10 (297.22–455.00)
Sex-adjusted HR (95% CI)	1.00(Referent)	0.64 (0.48–0.86)
Multivariable-adjusted ^a^ HR (95% CI)	1.00(Referent)	0.41 (0.28–0.58)

^a^: adjusted variables including age, SBP, BMI, LDL-C levels, current smoking, current drinking, antihypertensive drugs, education and physical activity

LDL-C: Low-density lipoprotein cholesterol. BMI: Body mass index. SBP: Systolic blood pressure

Education degree: Illiteracy and primary school, Junior high school and High school and above

Physical activity: Low, Moderate and high

Table 3 shows the single covariate analysis in Cox proportional hazard models. Firstly, we used the Cox proportional hazard models to examine the independent impact of sex. The HR of sex was 0.55 in the base model in the all stroke. When each potential factor was added to the base model, the range of HR changed from 0.55 to 0.50. Among the variables, the systolic blood pressure (SBP), current drinking and education degree produced the largest change in HR of sex (− 11.11% for each), followed by the antihypertensive drugs (− 8.89%), LDL-C levels and physical activity (− 2.22% for each). Smoking, age and BMI led to an increase in HR (6.67, 6.67, and 4.44% respectively).

**Table 3:** Contributions of each covariate in sex difference in incident stroke among hypertensive populations (single variable analysis)

Cox proportional hazard models	HR of sex (95% CI)	Percentage of sex difference accounted
All stroke		
Base model: sex (women/men)	0.55 (0.46–0.66)	
Base model+Age	0.58 (0.49–0.70)	6.67%
Base model+SBP	0.50 (0.42–0.59)	−11.11%
Base model+BMI	0.57 (0.47–0.68)	4.44%
Base model+Baseline LDL-C	0.54 (0.45–0.64)	−2.22%
Base model+Smoking	0.58 (0.48–0.71)	6.67%
Base model+Drinking	0.50 (0.41–0.62)	− 11.11%
Base model+Antihypertensive drugs	0.51 (0.43–0.61)	−8.89%
Base model+Education degree	0.50 (0.42–0.60)	−11.11%
Base model+Physical activity	0.54 (0.45–0.64)	−2.22%
Ischemic stroke		
Base model: sex (women/men)	0.48 (0.38–0.61)	
Base model+Age	0.51 (0.41–0.64)	5.77%
Base model+SBP	0.44 (0.35–0.55)	−7.69%
Base model+BMI	0.49 (0.39–0.61)	1.92%
Base model+Baseline LDL-C	0.46 (0.37–0.58)	−3.85%
Base model+Smoking	0.56 (0.43–0.72)	15.38%
Base model+Drinking	0.45 (0.34–0.58)	−5.77%
Base model+Antihypertensive drugs	0.44 (0.35–0.56)	−7.69%
Base model+Education degree	0.44 (0.35–0.56)	−7.69%
Base model+Physical activity	0.47 (0.37–0.59)	−1.92%
Hemorrhagic stroke		
Base model: sex (women/men)	0.64 (0.48–0.86)	
Base model+Age	0.67 (0.50–0.89)	8.33%
Base model+SBP	0.56 (0.42–0.75)	−22.22%
Base model+BMI	0.67 (0.50–0.89)	8.33%
Base model+Baseline LDL-C	0.64 (0.48–0.86)	0.00%
Base model+Smoking	0.57 (0.41–0.78)	−19.44%
Base model+Drinking	0.56 (0.40–0.79)	−22.22%
Base model+Antihypertensive drugs	0.58 (0.43–0.78)	−16.67%
Base model+Education degree	0.56 (0.42–0.75)	−22.22%
Base model+Physical activity	0.62 (0.47–0.83)	−5.56%

LDL-C: Low-density lipoprotein cholesterol. BMI: Body mass index. SBP: Systolic blood pressure. HR: Hazards ratios. CI: Confidence intervals

Education degree: Illiteracy and primary school, Junior high school and High school and above

Physical activity: Low, Moderate and High

Subgroup analysis shows that in ischemic stroke the results were accordance with the all stroke and current smoking was the primary influence factor (15.38%), the following major factors were SBP (− 7.69%), antihypertensive drugs (− 7.69%), education (− 7.69%), age (5.77%) and current drinking (− 5.77%); in hemorrhagic stroke the results were slightly different, SBP, current drinking and education were the major factors (− 22.22% for each), followed by the current smoking (− 19.44%), antihypertensive drugs (− 16.67%), age (8.33%), BMI (8.33%), and physical activity (− 5.56%). It is noteworthy that LDL-C levels were not change the HR of sex in our analysis.

Table 4 shows the multivariate analysis in Cox proportional hazard models. In the all stroke, when covariates such as age, SBP, BMI, LDL-C levels, current smoking, current drinking, antihypertensive drugs, education and physical activity were added to the base model step by step, the HR of sex changed from 0.55 to 0.44. In the complete model, including the above-mentioned variables, 24.44% of sex difference was accounted for and the HR of sex remained statistically significant.

**Table 4:** Contributions of each covariate in sex difference in incident stroke among hypertensive populations (multiple variable analysis)

Independent variables in Cox proportional hazard models	HR of sex (95% CI)	Percentage of sex difference accounted
All stroke		
Sex (base model)	0.55 (0.46–0.66)	
Variables in above model+Age	0.58 (0.49–0.70)	6.67%
Variables in above model+SBP	0.52 (0.43–0.62)	−6.67%
Variables in above model+BMI	0.52 (0.44–0.62)	−6.67%
Variables in above model+Baseline LDL-C	0.50 (0.42–0.60)	−11.11%
Variables in above model+Smoking	0.52 (0.43–0.63)	−6.67%
Variables in above model+Drinking	0.49 (0.39–0.60)	−13.33%
Variables in above model+Antihypertensive drugs	0.48 (0.39–0.59)	−15.56%
Variables in above model+Education degree	0.44 (0.35–0.55)	−24.44%
Variables in above model+Physical activity	0.44 (0.35–0.55)	−24.44%
Ischemic stroke		
Sex (base model)	0.48 (0.38–0.61)	
Variables in above model+Age	0.51 (0.41–0.64)	5.77%
Variables in above model+SBP	0.46 (0.37–0.58)	−3.85%
Variables in above model+BMI	0.46 (0.36–0.58)	− 3.85%
Variables in above model+Baseline LDL-C	0.43 (0.34–0.54)	−9.62%
Variables in above model+Smoking	0.49 (0.38–0.63)	1.92%
Variables in above model+Drinking	0.46 (0.35–0.60)	−3.85%
Variables in above model+Antihypertensive drugs	0.45 (0.34–0.59)	−5.77%
Variables in above model+Education degree	0.43 (0.32–0.56)	−9.62%
Variables in above model+Physical activity	0.43 (0.32–0.57)	−9.62%
Hemorrhagic stroke		
Sex (base model)	0.64 (0.48–0.86)	
Variables in above model+Age	0.67 (0.50–0.89)	8.33%
Variables in above model+SBP	0.57 (0.43–0.77)	−19.44%
Variables in above model+BMI	0.58 (0.44–0.78)	−16.67%
Variables in above model+Baseline LDL-C	0.59 (0.44–0.79)	−13.89%
Variables in above model+Smoking	0.50 (0.36–0.69)	−38.89%
Variables in above model+Drinking	0.48 (0.34–0.69)	−44.44%
Variables in above model+Antihypertensive drugs	0.47 (0.33–0.67)	−47.22%
Variables in above model+Education degree	0.41 (0.29–0.59)	−63.89%
Variables in above model+Physical activity	0.41 (0.28–0.58)	− 63.89%

LDL-C: Low-density lipoprotein cholesterol. BMI: Body mass index. SBP: Systolic blood pressure. HR: Hazards ratios. CI: Confidence intervals

Education degree: Illiteracy and primary school, Junior high school and High school and above

Physical activity: Low, Moderate and High

Subgroup analysis shows that these factors can explain 9.62% of the sex disparity in ischemic stroke and 63.89% of the sex disparity can be explained by these factors in hemorrhagic stroke.

## Discussion

In this study, we quantitatively estimate the factors for disparity in the incidence of stroke and its subtypes between women and men among hypertensive patients in rural areas of China. Our results showed that approximately 25% sex difference can be explained by the age, SBP, BMI, LDL-C levels, current smoking, current drinking, antihypertensive drugs, education and physical activity in the all stroke. In addition, this sex disparity in hemorrhagic stroke can be explained greater by those above-mentioned variables.

The present study demonstrated that the incidence of stroke was higher in men than that in women both for the all stroke and its subtypes, which was consistent with the previous studies [7, 9, 10, 22]. The stroke incidence was 33% higher in men than in women worldwide [7] and a systematic review showed that stroke incidence was about 30% higher in men than in women in Western Europe [10]. And the Tianjin Brain Study in China was also found that the age-standardized incidence of first-ever stroke was higher in men than in women [7].

Our results indicated that SBP, drinking and education were the primary contributors to the sex difference in the incidence of all stroke. Hypertension is a most common risk factor of stroke and previous studies indicated that BP values are higher in men than women of similar ages [10]. This difference may be due to the effect of the sex steroid hormones. Sex steroid hormones on the cerebral vasculature has the ability to alter vascular reactivity and thereby modulate blood flow and male arteries tend to be more constricted in response to pressure compared with arteries from females [23]. So men are suggested to take more attention to their BP values especially SBP values. Previous study showed that the association between alcohol intake and stroke morbidity and mortality was J-shaped [24]. And drinking was more common in men than that in women [8, 16]. We also observed a higher proportion of men drinkers in our study, and more men should be advised to abstain from alcohol. The former literature has reported that women are more likely to recognize at least 1 stroke warning sign than are men [25]. The lower education level was associated with increased stroke risk partially mediated by lifestyle and biological factors [7]. So it is demonstrating that educating the public particularly on men about the signs and symptoms of stroke may be key to preventing stroke deaths.

The effectiveness of antihypertensive drugs on CVD events and total mortality have been demonstrated [26, 27]. Former study reported that women in the youngest age group were less likely to be prescribed antihypertensive drugs, whereas in the oldest age group they were more likely to be prescribed antihypertensive drugs compared with their male counterparts [28]. In our study women have a higher proportion of antihypertensive medication use than men across all age groups (data not shown), which is slightly different with the former literature [28]. Thereby, more attention are recommended to focus on the men as the effectiveness of antihypertensive medication.

The higher level of LDL-C is associated with the increased risk of stroke and study indicates that women are more likely to have dyslipidemias [8]. And a research has shown that hypertriglyceridemia is associated with an increased risk of CVD mortality in women but not in men [29]. In our study, the average LDL-C levels were 0.14 mmol/L higher in women than in men. This phenomenon might in part be explained by the eating habits for women in China rural areas, such as prefer to the greasy food and read meat. For this reason, public are advised to take a healthy diet and pay more attention to their cholesterol levels, particularly men.

In our study physical activity also displayed a contribution to the sex difference in the incidence of all stroke. The protective effect of physical activity may be partly mediated by its role in reducing BP and controlling other risk factors for stroke, such as diabetes and excess body weight [30]. Physical activity affects men more than women may be due to the role of different risk factors between sex in our study.

**S**troke is a disease of elderly and several studies have demonstrated that the incidence of stroke was higher in older women and their outcomes are even worse compared to men [8–10]. The reason is that women are live longer and older women are more likely to live alone and lack of social support. In China, the mean age of people with incident stroke was 66.4 (SD 12.04) years: 65.5 (11.95) years in men and 67.6 (12.05) years in women [6]. Compared with it, our incident stroke cases were younger. The mean age was 60.4 (10.99) years: 61.5 (10.83) years in men and 59.0 (11.05) years in women (data not shown). It is demonstrating that stroke in rural areas of China northeast was more severe. Therefore, the corresponding policy and intervention from the government and the health care sector should be strengthen. Smoking is an independent risk factor for stroke in both sexes [31]. Sanne A.E. Peters’ study indicated that women have a more harmful effect of smoking than men in Western populations but not in Asian populations [31]. Another study found that women have greater excess risk of CVD from heavy smoking than men in the Asia-Pacific region [32]. It is consistent with our study and this may be due to the tobacco use is spreading among women and the harmful effect of second-hand cigarette. Thereby, tobacco control policies should emphasize the specificity of women and the importance of controlling tobacco especially in the public place. Previous studies have illuminated that higher BMI levels were more common in women than that in men [7, 8], which was consistent with our study. In recent years, the prevalence of obesity is rising in China as the transitions of lifestyle, so some chronic diseases become more prevalent. BMI increase the risk of stroke and the most important three mediators are high BP, cholesterol, and glucose [33]. So control weight in reasonable context, in particular for women, are necessary.

In addition, the single variable analysis of ischemic stroke presented coincident trend with the all stroke and smoking was the primary contributor. In hemorrhagic stroke, however, we observed a slightly difference compared to the all stroke. First, smoking showed a reverse relationship with hemorrhagic stroke compared to the all stroke. Second, we can found that LDL-C levels were not change the HR of sex. This phenomenon may be associated with the physiopathologic mechanisms of hemorrhagic stroke. In the multivariate analysis of ischemic stroke and hemorrhagic stroke, we can found a remarkable decrease in HR of sex in hemorrhagic stroke than in ischemic stroke, this suggested that hemorrhagic stroke can be greater controlled by these variables.

Strengths of our study include the relatively large sample size and relatively long period of follow-up, generate a large number of stroke cases and increase the statistical power. Some limitations should also be considered in light of these results. Firstly, our study was restricted to a country sample in northeast China, which limits the generalization of our results, more diverse population are needed to confirm our results. Secondly, there may be some minor stroke cases were not recorded as a result of they not seeking emergency care so the stroke cases may be underestimated. Thirdly, there may be some other factors that we did not recorded can affect the sex difference in stroke incidence, such as the genetic factors, and further research would be encouraged.

## Conclusion

Our results indicate that the stroke incidence varied by sex and men had a higher incidence than that in women. This difference was partly explained by several traditional cardiovascular risk factors. The remaining differences need to be explained by other factors, such as genetic factors.

## Abbreviations

BMI: Body mass index
BP: Blood pressure
CAD: Coronary artery disease
CI: Confidence interval
CVD: Cardiovascular disease
HDL-C: High-density lipoprotein cholesterol
HR: Hazard ratios
LDL-C: Low-density lipoprotein cholesterol
SBP: Systolic blood pressure
SD: Standard deviation
